# Structure based discovery of clomifene as a potent inhibitor of cancer-associated mutant IDH1

**DOI:** 10.18632/oncotarget.17464

**Published:** 2017-04-27

**Authors:** Mengzhu Zheng, Weiguang Sun, Suyu Gao, Shanshan Luan, Dan Li, Renqi Chen, Qian Zhang, Lixia Chen, Jiangeng Huang, Hua Li

**Affiliations:** ^1^ Hubei Key Laboratory of Natural Medicinal Chemistry and Resource Evaluation, School of Pharmacy, Tongji Medical College, Huazhong University of Science and Technology, Wuhan 430030, China; ^2^ Wuya College of Innovation, School of Traditional Chinese Materia Medica, Key Laboratory of Structure-Based Drug Design & Discovery, Ministry of Education, Shenyang Pharmaceutical University, Shenyang 110016, China; ^3^ Department of Mathematics Computer Science, Emory College of Undergraduates, Emory University, Atlanta, GA 30322, USA

**Keywords:** IDH1, clomifene, virtual ligand screening, cancer, drug repurposing

## Abstract

Isocitrate dehydrogenase (IDH) plays an indispensable role in the tricarboxylic acid cycle, and IDH mutations are present in nearly 75% of glioma and 20% of acute myeloid leukemia. One IDH1R132H inhibitor (clomifene citrate) was found by virtual screening method, which can selectively suppress mutant enzyme activities *in vitro* and *in vivo* with a dose-dependent manner. The molecular docking indicated that clomifene occupied the allosteric site of the mutant IDH1. Enzymatic kinetics also demonstrated that clomifene inhibited mutant enzyme in a non-competitive manner. Moreover, knockdown of mutant IDH1 in HT1080 cells decreased the sensitivity to clomifene. *In vivo* studies indicated that clomifene significantly suppressed the tumor growth of HT1080-bearing CB-17/Icr-scid mice with oral administration of 100 mg/kg and 50 mg/kg per day. In short, our findings highlight clomifene may have clinical potential in tumor therapies as a safe and effective inhibitor of mutant IDH1.

## INTRODUCTION

Isocitrate dehydrogenases (IDH) are a family of enzymes that catalyze the conversion of isocitric acid (ICT) to α-ketoglutaric acid (α-KG) which is one of the key reactions in the tricarboxylic acid cycle [1, 2]. This enzymatic pathway is associated with various molecular processes regulating the cellular epigenetic states, including histone and DNA modifications. Accumulated evidences have revealed mutant IDH enzymes are observed in multiple human cancers. Reportedly, among the three isoforms of IDH in humans, frequent somatic mutations of IDH1 have recently been found in certain cancers including nearly 80% in grade II-III gliomas, appropriate 45% in secondary glioblastoma multiforme (GBM), and 33%-50% in adult primitive neuroectodermal tumor [3, 4]. Besides brain tumors, IDH1 mutations have also been detected in other cancers including acute myeloid leukemia [5], colorectal cancer [6], and prostate cancer with low frequencies [7].

Biochemically, all of the mutant IDH proteins, including IDH1R132H and IDH1R132C, concomitant gain of a neomorphic function that reduce α-KG to D-2-hydroxyglutaricacid (D-2HG) using NADPH as the cofactor [8]. D-2HG has been confirmed to be an inhibitor of α-KG-dependent dioxygenases, therefore, as a result of mutations in IDH, high cellular concentration of D-2HG may cause global methylation of histone and DNA, which may lead to tumorigenesis [9]. In summary, these findings suggested that novel specific inhibitors of mutant IDH1 may become a new therapy for glioma, AML and other cancers with IDH1 mutation.

Nowadays, structure-based virtual ligand screening is playing an increasingly important role in early-stage drug discovery [10]. In this study, we conducted a structure based virtual ligand screening to identify small molecule inhibitors of mutant IDH1. Followed by further examination and verification, we confirmed clomifene, an effective and low-cost medication for ovulation induction, as a novel inhibitor of mutant IDH1, which could reduce the cellular and tumor levels of D-2HG, showing dramatically on-target activity both *in vitro* and *in vivo*.

## RESULTS

### Virtual ligand screening identifies the binding of clomifene to mutant IDH1

The X-ray structure of the IDH1R132H heterodimer (PDB: 4UMX) [11, 12] was chosen to generate a molecular model for our investigations. The non-commercial database, ZINC Drug Database (ZDD) containing over 2924 approved drugs and nutraceuticals were screened against this model in silicon by ICM-Pro 3.8.1 molecular docking software (Molsoft LLC, San Diego, CA) [13]. Compounds with lower calculated binding energies were considered to have higher binding affinities with the target. The docking score results were presented in Table 1. There were ten compounds showing significant ICM scores (< -27) which predicted their possible bindings to mutant IDH1 heterodimer (Supplementary Figure 1). The proposed binding site and binding pose of compounds were inspected and results were listed in the Table 1. Clomifene, cefoxitin sodium, tigecycline and adapalene were predicted to coordinate the binding pocket of the ligand in the crystal structure with high occupancy.

**Table 1:** Predicted binding free energies and inhibitory activities of screening hits

Compound	ICM scores^a^	Binding Pocket^c^	Kd (*μ*M)^b^	IC_50_ for enzyme activities (*μ*M)		
				WT	R132H	R132C
Clomifene	−29.88	5/6, Y	18.45 ± 1.61	>200	50.20 ± 0.12	42.33 ± 0.31
Cefoxitin sodium	−33.53	2/3, Y	n.b^d^	>200	>200	>200
Rivaroxaban	−31.33	1/2, Y	103.11 ± 11.32	>200	>200	>200
Dasatinib	−31.38	1/2, Y	28.71 ± 2.48	>200	>200	>200
Demeclocycline	−32.89	1/2, Y	303.00 ± 3.15	n.p^e^	n.p	n.p
Metacycline	−37.48	N	n.b	n.p	n.p	n.p
Fluconazole	−33.33	N	n.b	n.p	n.p	n.p
Tigecycline	−32.24	2/3, Y	n.b	n.p	n.p	n.p
Propafenone Hydrochloride	−32.44	1/2, Y	n.b	n.p	n.p	n.p
Adapalene	−27.23	5/6, Y	n.b	n.p	n.p	n.p
AGI-5198	−20.18	2/3, o.p^f^	2.90 ± 0.14	>200	5.70 ± 0.24	10.62 ± 0.63

^a^ Docking score/interaction potential of compounds with IDH1R132H (kcal/mol).

^b^ The Kd value is automatic calculated by the curve fitting, and presents as means ± SD for three experiments.

^c^ Whether compounds were docking into the binding pocket of the ligand and their occupancy; Y, yes; N, no; the fractions indicate the occupying rate.

^d^ n.b is no clear binding detected in the MST measurement.

^e^ n.p is no clear inhibitory detected.

^f^ o.p means other pocket. There are two different binding pockets in this molecular docking model as presented in Supplementary Figure 4.

### Specific binding of clomifene with mutant IDH1

**Figure 1:**
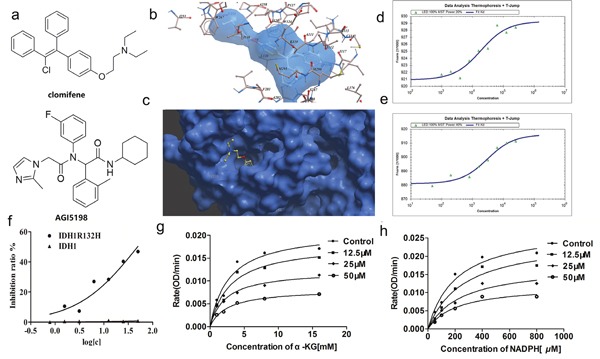
Clomifene specifically binds with IDH1R132H and selectively inhibits mutant IDH1 activity *in vitro* **(a)** Chemical structure of clomifene and AGI-5198. **(b)** Detailed view of clomifene binding in the ligand binding pocket. **(c)** Overlay of one monomer of the IDH1 R132H-clomifene binary complex. **(d, e)** Measurements of affinity of clomifene and AGI5198 with IDH1R132H by MST in standard treated capillaries, the resulting binding curve was shown. From the binding curve, the Kd values of 18.45 ± 1.61 *μ*M (Clomifene) and 2.90 ± 0.14 *μ*M (AGI5198) were calculated. **(f)** IC_50_ of clomifene against wild-type and mutant IDH1 and IDH1 **(g, h)** Clomifene inhibits IDH1R132H noncompetitively with respect to NADPH and α-KG, respectively.

Microscale thermophoresis (MST) method was further employed to validate the finding of virtual screening. MST technology is based on fluorescence detection and thermophoresis to precisely measure protein-protein and protein-small molecule interactions with low sample consumptions [14]. Of all the compounds assaied, clomifene exhibited the lowest equilibrium dissociation constant (Kd) of 18.45 ± 1.61 *μ*M (Table 1, Figure 1a and 1d), which means the strongest binding with the target protein. The binding affinity of clomifene was comparable to that of AGI-5198, a previously reported IDH1R132H inhibitor (Table 1, Figure 1a and 1e). Results of MST measurements proved this hypothesis that clomifene was the one showing highest binding affinity to the mutant IDH1 (Supplementary Figure 2).

Further enzyme inhibition assay confirmed clomifene as the only inhibitor of mutant IDH1 with reasonable IC_50_ among 10 compounds (Table 1).

### Binding mode of clomifene with IDH1R132H predicted by molecular docking

In order to further elucidate the binding mode of clomifene with mutant IDH1, molecular docking was carried out. The lowest-energy binding conformation of clomifene was shown as Figure 1b and 1c. From the generated docking model, clomifene well fitted the ligand binding site of mutant IDH1 and almost completely occupied the whole pocket with an extended conformation. The ligand binding pocket of mutant IDH1 was gourd shaped, where many hydrophobic amino acid, including Ile112, Ile117, Leu120, Trp124, Ile128, Ile130, Val255, Trp267, Val281, Tyr285, Leu288, Met290 and Leu376, form a highly hydrophobic envelop. Clomifene formed obvious hydrophobic interactions with many of these amino acids, like two ethyl groups with Val255 and Trp267, benzene ring of phenoxy with Trp124, two phenyls with Tyr285, 1-phenyl with Met291. Although there was no hydrogen bonding formed between clomifene and mutant IDH1 protein, seemingly highly hydrophobic clomifene molecule could form enough interactions with the equally hydrophobic binding pocket.

### Clomifene selectively inhibits mutant IDH1 activity *in vitro*

In order to identify the selective inhibitory activity of screening hits against IDH1R132H, IDH1R132C and wild type (WT) IDH1, recombinant proteins of all constructs were overexpressed and purified. Enzyme activity assay demonstrated that clomifene inhibited IDH1R132H and IDH1R132C with IC_50_ values of 50.20 ± 0.12 *μ*M and 42.33 ± 0.31 *μ*M but did not obviously alter the viability of WT IDH1 even at 200 *μ*M (Table 1, Figure 1f), which indicated the reasonable selectivity and significant inhibitory of clomifene.

The inhibition of IDH1R132H by clomifene was showing a non-competitive manner for the substrate α-KG and NADPH (Figure 1g and 1h). Initial kinetic studies indicated that the Vmax of mutant IDH1 was 0.065 OD/min and the Km for α-KG was 2.792 mM, after treatment with 50 *μ*M clomifene, the Km and Vmax calculated from Michaelis-Menten curve changed to 2.571 mM and 0.025 OD/min respectively (Supplementary Table 1). Molecular docking predicted that clomifene bind with mutant IDH1 in the ligand binding pocket, in which the ligand, 2,6-bis (1H-imidazol-1-ylmethyl)-4-(2,4,4-trimethylpentan-2-yl) phenol, a non-competitive and allosteric inhibitor bind with the enzyme in the allosteric site [10]. The result of enzymatic inhibition assay which was showing non-competitive manner was consistent with the prediction of docking and results of MST binding assay.

### Clomifene inhibits mutant IDH1 activity and represses cancer cell growth *in vitro*

**Figure 2:**
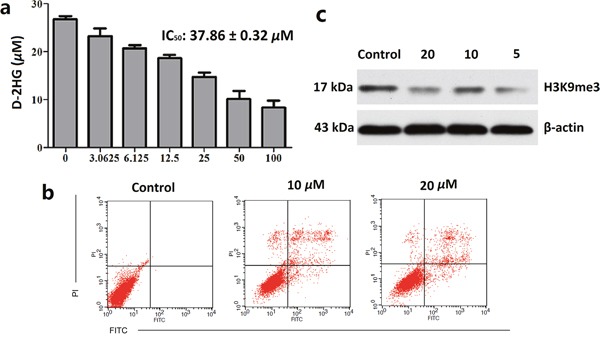
Clomifene suppresses IDH1 activity in celluro **(a)** Inhibitory activities of IDH1R132H enzyme lead to decreased production of D-2HG in IDH1R132H mutant HT1080 cells after treatment with clomifene for 48 h, and D-2HG was measured by LC/MS method. **(b)** HT1080 cells were treated for 24 h in the different concentrations of clomifene and then processed for FACS by using Annexin V/propidium iodide staining. **(c)** Treatment of clomifene significantly reduced methylation levels of histone lysine residues in HT1080 cells. All data are the average of results from triplicate experiments.

Encouraging by promising inhibitory activities of clomifene on mutant IDH1 enzymes activity, in consideration of both IDH1R132H and IDH1R132C concomitant gain of the same neomorphic function that reduce α-KG to D-2-hydroxyglutaricacid (D-2HG) using NADPH as the cofactor, human fibrosarcoma cell line HT1080 harboring R132C mutation of IDH1, the most common IDH mutation in glioma, was selected to further examined for its biological activity *in vitro* [15]. After treatment with various concentrations of clomifene or 0.5% DMSO as a vehicle control for 48 h, the inhibition of mutant IDH1 was tested by measuring the concentration of D-2HG in cellular supernatant via a LC-MS method. As shown in Figure 2a, a dose-dependent decrease in D-2HG levels with an IC_50_ value of 37.86 ± 0.32 *μ*M was observed.

Effects of clomifene on the cellular apoptosis of HT1080 cells were further investigated using flow cytometry analysis. Normalization to the DMSO-treated controls revealed that clomifene at 10 *μ*M and 20 *μ*M induced 12.88%, and 23.61% apoptotic death compared with 2% of the control (Figure 2b).

To further understand the mechanism of clomifene-induced cell growth inhibition and apoptosis, we next investigated whether the histone methylation in cells could be affected. As mentioned above, D-2HG is an inhibitor of histone demethylases, which are α-KG-dependent dioxygenases [16]. Consequently, D-2HG may cause global methylation of histones. After treated with clomifene for 48 hours, histone proteins were extracted and methylation level of H3K9me3 was measured by western blot analysis [17]. As shown in Figure 2c, clomifene could markedly decrease the level of histone methylation in HT1080 cells with a dose-dependent manner. These results suggested that clomifene could restore the activities of histone demethylases and avoid the wide hypermethylation of histones, as the result of reducing the concentration of D-2HG.

### Knocking down IDH1R132C attenuates the inhibitory effect of HT1080 cell growth by clomifene

**Figure 3:**
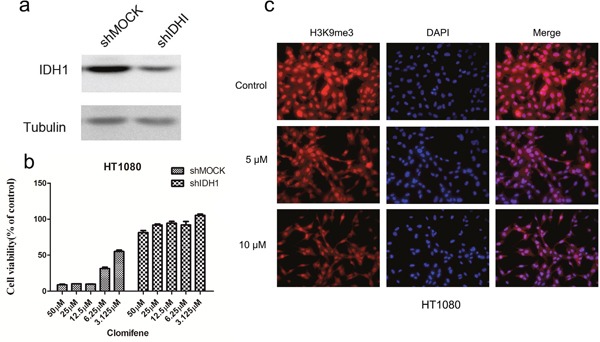
Knocking down IDH1R132C attenuates the inhibitory effect of HT1080 cell growth by clomifene **(a)** Expression level of IDH1 in HT1080 cells is decreased by knockdown of IDH1. HT1080 cells were transiently transfected with shMOCK or shIDH1 and cell lysates were analyzed by western blot. **(b)** Clomifene has less effect on cell growth of shIDH1 transfected cells than that of shMOCK cells. **(c)** Mutant IDH1-mediated H3K9me3 was substantially decreased dose dependently with clomifene treatment.

In addition, to investigate whether the effects of clomifene were mediated directly through mutant IDH1, we compared effects of HT1080 cells transfected with a shMOCK or shIDH1R132C plasmid (Figure 3a). Clomifene suppressed growth of shMOCK cells but had fewer effects on shIDH1R132C cells (Figure 3b). Immunocytochemical staining results showed that mutant IDH1-mediated H3K9me3 was substantially and dose-dependently decreased with clomifene treatment (Figure 3c). These results suggested that mutant IDH1 was a direct target for clomifene to suppress cancer cell growth.

### Clomifene suppresses tumor growth by inhibiting mutant IDH1 activity *in vivo*

All above tests and data showed clomifene was an effective inhibitor of IDH1R132H *in vitro*. We next evaluated the *in vivo* antitumor efficacy of clomifene. In the xenograft model, HT1080 cells were inoculated subcutaneously into the right flank of male CB-17/Icr-scid mice [18]. The tumor-bearing mice were then divided into three matched groups including the vehicle control and two clomifene treating groups (50, 100 mg/kg). After establishing palpable tumors, mice were administrated with clomifene or vehicle via gavage once every day.

**Figure 4:**
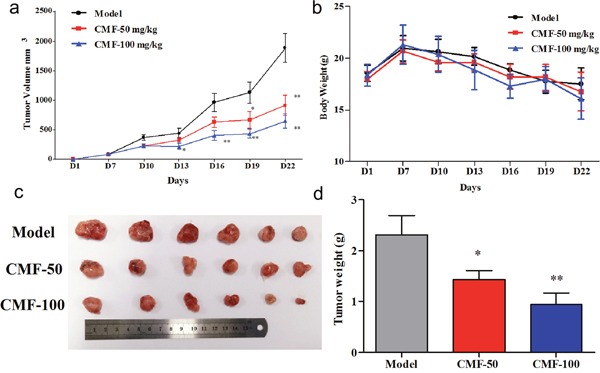
Effect of Clomifene(CMF) on fibrosarcoma cancer growth in an HT1080 xenograft mouse model **(a)** The average tumor volume of vehicle-treated control mice (n = 6) and clomifene treated mice (n = 6, 50 or 100 mg/kg per day *via* gavage) plotted over 21 days after tumor cell injection. **(b)** Clomifene has no effect on mouse body weight. **(c, d)** After 2 weeks’ treatment, differences in tumor size and weight are shown. The asterisk * indicates a significant increased tumor size (*P* < 0.05) in the vehicle-treated group compared with the clomifene-treated group as determined by one-way analysis of variance.

Estimated tumor volumes of both treating groups were much less than that of the control group throughout the therapeutic process. (Figure 4a, Figure 4c, Supplementary Table 2). After 2 weeks’ treatment, all mice were then scarified and the average tumor weights were calculated. The tumor weight of the control group was 2.32 ± 0.93 g, whereas that of low-dose and high-dose treated groups were 1.43 ± 0.42 g and 0.94 ± 0.61 g respectively (Figure 4d, Supplementary Table 3), representing significant inhibition ratio. Moreover, no obvious hepatotoxicity and nephrotoxicity, or different of average body weights between three groups were observed throughout the study (Figure 4b, Supplementary Figure 3), which revealed that the dosage of clomifene used in this experiment had no obvious side effects on animals’ growth. Furthermore, clomifene at 100 mg/kg decreased the tumor weight by about 60% (Supplementary Table 3), comparable to the results of AGI-5198 at a dose of 450 mg/kg [4].

**Figure 5:**
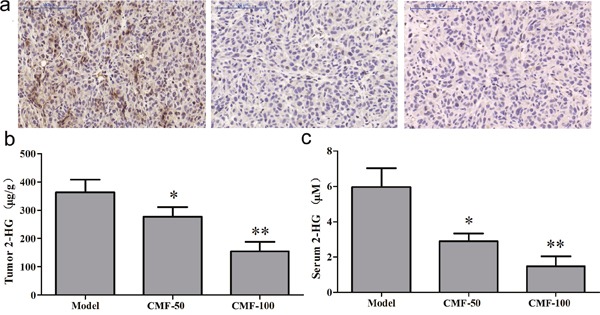
Clomifene (CMF) suppresses IDH1 activity *in vivo* **(a)** Immunohistochemical analysis for the H3K9me3 antibodies was analyzed using mouse tumor samples. The magnification of representative photographs for the immunohistochemistry staining is 400×. **(b, c)** Clomifene inhibits D-2HG production in serum and tumor tissues.

We then examined D-2HG concentrations in tumors and serum. As shown in Figure 5b, D-2HG concentration of tumour tissue from vehicle control group was 364.01 ± 118.43 *μ*g/g (Supplementary Table 4). By normalization to the control group, clomifene at 50 and 100 mg/kg could induce 23.81% and 57.38% inhibitory of D-2HG production respectively, meanwhile, a similar inhibition was detected in serum (Figure 5c, Supplementary Table 4). In order to further reveal the relationship between antitumor effects and inhibitions of mutant IDH1 enzymes by clomifene, tumor tissues extracted from different groups were subjected to an immunohistochemistry analysis for H3K9me3. Results exhibited that expressions of H3K9me3 were substantially reduced in clomifene treated groups compared with the vehicle group (Figure 5a). Taken together, these results suggested that clomifene effectively and safely suppresses the tumor growth *in vivo* by inhibiting mutant IDH1.

## DISCUSSION

New drug development is a time-consuming and costly process, and the discovery of a new chemical entity need to pay a lot of cost, many candidate compounds failed in the late phase of clinical trials. Drug repurposing is able to partly overcome the difficulties of this process, because a known drug has been widely used in clinical practice; its druggability and safety have been verified. What you need is to change the indication by demonstrating its pharmacological effects on the new indication. Therefore, this method is becoming an effective way to discover new drugs rapidly and has been widely applied in recent years.

Structure based virtual screening represents a new direction for drug discovery and has been gradually developed recently, by the method, one can quickly screen large numbers of compounds in a short period of time, to find possible candidate compounds, with low cost and high success rate. Applying the virtual screening method to drug repurposing by screening drug database based on the target structure is an effective way to discover new indications of old drugs, and there are many successful examples in recent years [19].

Clomifene was used as a selective estrogen receptor modulator for treatment of female infertility due to anovulation [20–22]. It was also proposed as a drug to reverse impotence in men due to low testosterone levels [23, 24] and is currently used off-label for men with hypogonadism. In this study, we identify clomifene as a potent mutant IDH1 inhibitor by structure based virtual ligand screening. In the crystal structure of inhibitor-mutant IDH1 complex (PDB code: 4UMX), which was chosen as the model for molecular docking, the non-competitive and allosteric ligand 2,6-bis (1H-imidazol-1-ylmethyl)-4-(2,4,4-trimethylpentan-2-yl) phenol bound with the enzyme in the allosteric site. From the generated docking model, clomifene was well fitted into the same allosteric site with an extended conformation. The inhibition of IDH1R132H by clomifene also showed a non-competitive manner for the substrate α-KG and NADPH, which was consistent with the results of molecular docking. Further molecular docking predicted that the binding site of AGI-5198 with mutant IDH1 was close to the pocket of active center near NADP (Supplementary Figure 4). Detailed studies of kinetic mechanism of action demonstrated that AGI-5198 played roles as competitive inhibitor with respect to α-KG and uncompetitive with respect to NADPH [8]. Thus, clomifene inhibits the mutant IDH1 with a different mechanism other than AGI-5198.

Previous studies have demonstrated that IDH1 mutations could catalyze another new reaction in which α-KG is reduced to D-2-hydroxyglutaricacid (D-2HG) using NADPH as the cofactor, which may cause global DNA and histone hypermethylation, and finally lead to carcinogenesis. Following biochemical verification, we confirmed that treatment with clomifene could strongly reduce productions of D-2HG *in vitro* and *in vivo*. Notably, clomifene markedly repressed the tumor growth in the mouse xenograft model accompanied by significantly reduced levels of H3K9me3 mark in tumor tissues.

In summary, we have identified a new inhibitor of mutant IDH1 from ZDD database by virtual ligand screening and demonstrated its pharmacological inhibition on mutant enzyme both *in vitro* and *in vivo*. Overall, our findings provide a possible drug candidate targeted mutant IDH1 and a new lead compound for IDH1 inhibitor discovery. As far as we know, clomifene is the first FDA approved drug reported to exhibit potent inhibitory activities against mutant IDH1 both *in vitro* and *in vivo*. This may accelerate the application of IDH1 inhibitors in clinical by applying new indications of this drug, to benefit a large number of patients with glioma or AML. In our future study, more detailed action mechanisms of clomifene will be studied, and more derivatives of clomifene will be synthesized followed by structure guided lead optimization.

## MATERIALS AND METHODS

### Reagents and antibodies

The HT1080 fibrosarcoma cell line harboring IDH1 (R132C) mutation (ATCC # CCL-121™) and WI-38 (ATCC # CCL-75™) were obtained from the American Type Culture Collection (ATCC, USA). Clomifene citrate was purchased from Selleck Chemicals (Huston, USA). The structure was confirmed by NMR and purity (99%) was measured by HPLC analysis. The antibody against H3K9me3 was obtained from Cell Signaling Technology (Beverly, MA), β-actin and secondary antibody from rabbit were purchased from Santa Cruz Biotechnology (Santa Cruz, CA). All other common chemicals, solvents and reagents were of highest grade available from various commercial sources.

### Molecular docking and virtual screening

First, the subset ZDD (ZINC Drug Database), a special subset of ZINC containing all commercially available approved drugs and nutraceuticals world wide, was downloaded from ZINC as mol2 files. Then molecular docking and virtual screening was performed on the compounds of the subset ZDD downloaded before. The docking was performed by using ICM 3.8.1 modeling software on an Intel i7 4960 processor (MolSoft LLC, San Diego, CA). Ligand binding pocket residues were selected by using graphical tools in the ICM software, to create the boundaries of the docking search. In the docking calculation, potential energy maps of the receptor were calculated using default parameters. Compounds were imported into ICM and an index file was created. Conformational sampling was based on the Monte Carlo procedure, and finally the lowest-energy and the most favorable orientation of the ligand were selected [25].

### Expression and purification of R132H and R132C mutant IDH1

R132H and R132C mutant IDH1 genes were generated from the wild-type IDH1 plasmid, using Quick-change site-directed mutagenesis kit (Agilent) following the manufacturer's protocol. Correctness of the gene sequences was verified. The IDH1R132H and IDH1R132C were cloned into the pET28a vector (Novagen). The expression construct pET28a-IDH1R132H and pET28a-IDH1R132HC were transformed into Escherichia coli strain BL21 (DE) (Invitrogen) and selected on kanamycin plates. The transformed cells were cultivated in Luria-Bertani (LB) media at 37 °C in the presence of kanamycin until the optical density (OD) reached 0.8. Cells were then induced with 0.4 mM IPTG (isopropyl-*β*-*D*-1-thiogalactopyranoside) for 16 h at 20 °C. The cells harvested by centrifugation were lysed by ultrasonication on ice in a buffer containing 20 mM Tris, pH 8.5, 200 mM NaCl, 5 mM mercaptoethanol, 0.1% TritonX-100, and 5% glycerol.

Soluble C-terminally hexa-histidine tagged IDH1R132H and IDHR132C were bound to Ni-agarose affinity resin (Qiagen), washed with buffer A (20 mM Tris, pH 8.5, 200mM NaCl, and 10 mM imidazole), and eluted with buffer B (20 mM Tris, pH 8.8, 250 mM NaCl, and 150 mM imidazole). The protein was further purified with anion exchange chromatography and size exclusion chromatography.

### Microscale thermophoresis (MST) assay

Recombinant IDHR132H was labeled with the Monolith NT™ Protein Labeling Kit RED (Cat # L001) according to the supplied labeling protocol. The concentration of labeled IDHR132H in the test was 50 nM. Samples were diluted in a 20mM HEPES (pH 7.5) and 0.5 (v/v) % Tween-20. The clomifene stock was dissolved in 10% DMSO at a concentration of 5 mM. We used 500 *μ*M clomifene as the highest concentration for the serial dilution. After 10 min incubation at room temperature the samples were loaded into Monolith™ standard-treated capillaries and the thermophoresis was measured at 25 °C after 30 min incubation on a Monolith NT.115 instrument (NanoTemper Technologies, München, Germany). Laser power was set to 40% using 30 seconds on-time. The LED power was set to 100%. The dissociation constrant Kd values were fitted by using the NTAnalysis software (NanoTemper Technologies, München, Germany)

### *In vitro* enzyme inhibition assay

The activity and inhibition of IDH1R132H and IDH1R132C were determined by measuring the initial linear consumption of NADPH of the reaction. The enzyme activity assay was performed in a 96-well microplate using the purified IDH1 mutant (2 *μ*M), 4 mM MgCl_2_, 2 mM α-KG, 100 *μ*M NADPH (>>Km for NADPH) in 50 mM HEPES buffer (pH = 7.5) containing 0.1 mg/mL bovine serum albumin. For inhibition assay, firstly, triplicate samples of compounds were incubated with the protein for 5 min. Next, the reaction was initiated by adding α-KG into the 96-well microplate. The consumption of NADPH was measured by monitoring the optical absorbance of each well every 30s at 340 nm, which is the maximum absorption wavelength of NADPH, using a BioTek Synergy HT microplate reader. The activity and inhibition of WT-IDH1 were determined by measuring the production of NADPH. In brief, the enzyme activity assay was performed in a 96-well microplate using the purified IDH1 (43 nM), 4 mM MgCl_2_, 50 *μ*M sodium (D)-isocitrate, 1mM NADP+ (>>Km for NADP) in 50 mM HEPES buffer (pH = 7.5). The reaction can be readily monitored by an increase in optical absorbance at 340 nm. The data were imported into Prism (version 6.02, GraphPad) and the IC_50_ values were calculated by using a standard dose response curve fitting.

### Cell culture

HT1080 and WI-38 cells were both cultured in high Glucose Dulbecco's modified Eagle's medium (DMEM) supplemented with 2 mM glutamine, 10% (v/v) fetal bovine serum (FBS), 100 U/mL penicillin and 100 mg/mL streptomycin. Cell cultures were grown and maintained in culture at 37 °C in a humidified tissue culture incubator with 5% CO_2_. The cell cultures were performed following the instructions of ATCC.

### Cytotoxicity test

To estimate cell viability, HT1080 and WI-38 cells (5000/well) were separately seeded in 96-well plates for 24 h at 37 °C in a 5% CO_2_ incubator. The attached cells were fed with fresh medium containing various concentrations of clomifene (0-100 *μ*M) for additional 48 h. After culturing for various times, the cytotoxicity of clomifene was measured using a CCK8 assay kit according to the manufacturer's instructions. All the experiments were performed in triplicate, and the mean absorbance values were calculated. The results are expressed as the percentage of inhibition that produced a reduction in absorbance by clomifene treatment compared with the non-treated cells.

### D-2HG Production

The D-2HG production inhibition assay was described as follows. In brief, 5000cells/well were seeded into wells of a 96-well plate for 24 h. Cells were then treated with an increasing concentration of compounds in 100 *μ*L of culture medium for 48 h. Medium was collected and diluted with 400 *μ*L of methanol. After shaking, the mixture was centrifuged to remove any precipitate, and the supernatant was prepared to make measurement of D-2HG by HPLC-MS. The LC-MS/MS system consisted of a Shimadzu Prominence UFLC system (Shimadzu Corporation, Kyoto, Japan) and an API4000 QTrap® triple quadrupole mass spectrometer (AB Sciex, Foster City, CA, USA) equipped with an electrospray ionization (ESI) source. Chromatographic separation was carried out on a Welch Ultimate® XB-C18 column at 40 °C (100 mm × 2.1 mm, 3.0 *μ*m; Welch Materials, Shanghai, China) protected by a C18 guard column (4.0 mm × 2.0 mm, 5 *μ*m; Phenomenex, Torrance, CA, USA). The mobile phase consisted of 10 mM ammonium acetate in water (pH = 7.4 adjusted with ammonium hydroxide, phase A) and methanol (phase B). The following gradient conditions were used: 0-1.50 min, 30-90% B; 1.50-2.00 min, 90% B; 2.00-2.01 min, 90-30% B; 2.01-3.00 min, 30% B. Mass spectrometric detection was performed in negative ion electrospray mode with multiple reactions monitoring (MRM), monitoring the precursor to product ion transition of m/z 146.8→128.6 for D-2HG. The source parameters were optimized as follows: curtain gas: 20 psi, collision gas (CAD): high, ionSpray voltage: -4500 V, temperature: 500 °C, nebulizer gas: 40 psi, auxiliary gas: 50 psi and interface heater: on. The optimum compound dependent parameters including decluttering potential (DP), collision energy (CE), entrance potential (EP) and cell exit potential (CXP) were set at -55, -14, -10, -9V for D-2HG. Data acquisition and processing were controlled by Analyst 1.6.1 software (AB Sciex, Foster City, CA, USA). Prior to measurements of D-2HG concentrations, the LC-MS/MS assay was validated and calibrated by authentic D-2HG purchased from Sigma Aldrich (St Louis, MO, USA). IC_50_ was calculated from the dose-response curve by Prism 6.02 [8].

### Flow cytometric analysis for apoptosis

HT1080 cells were cultured in six-well plates and treated with different concentrations of clomifene for 48 h. Then the cells were harvested, washed twice with ice-cold PBS, and mixed in 100 *μ*L of 1 × binding buffer (10 mM HEPES/NaOH, pH 7.4, 140 mM NaCl, 2.5 mM CaCl_2_). After culturing for 15 min at room temperature in Annexin-V/PI (Nanjing KeyGen Biotech. Inc.) double staining liquid, the cells were examined by flow cytometry (BD Biosciences, FACSCalibur).

### Western blot analysis

The harvested cells were lysed with lysis buffer (50 mM Tris-HCl (pH 7.4), 150 mM NaCl, 1 mM EDTA, 1 mM EGTA, 10 mg/mL aprotinin, 10 mg/mL leupeptin, 5 mM phenylmethanesulfonyluoride (PMSF), 1 mM dithiolthreitol (DTT) containing 1% Triton X-100). Insoluble debris was removed by centrifugation at 13000 rpm for 20 minutes, and the content of protein was determined using Bradford reagent (Bio-Rad, USA). Lysate protein (20-40 *μ*g) was subjected to 12% SDS-PAGE and electrophoretically transferred to polyvinylidene difluoride membranes (PVDF) (Millipore, USA). The membranes were blocked with 5% non-fat milk for 1 h and then incubated with the respective specific primary antibody at 4 °C overnight. Protein bands were visualized using an enhanced chemiluminescence reagent (ECL Plus) (GE Healthcare, USA) after hybridization with a HRP conjugated secondary antibody. Band density was quantified using the Image J software program (NIH).

### Generation of sublines with inducible IDH1 knockdown

HT1080 cells were infected with lentiviral particles containing either pTRIPz empty vector (OpenBiosystems), pTRIPz IDH1 sh484 (targeting the CDS). The target sequences of oligo siRNAs were as follows: 5'-GGACTTGGCTGCTTGCATT-3' for IDH1 to produce lentiviral particles, 293T cells were co-transfected with pTRIPz vectors, psPAX2 (Addgene # 12260), and pMD2.G (Addgene # 12259) and supernatant containing viral particles was collected at 36 and 72 hours [16].

### Immunocytochemical staining

We seeded the cells at 2×10^5^ per well (Lab-Tek II chamber slide, Nalgen Nunc International, Naperville, IL). 48 hours after incubation, cells were fixed with PBS (−) containing 4% paraformaldehyde for 15 minutes, and rendered permeable with PBS (−) containing 0.1% Triton X-100 at 4 °C for 2.5 minutes. Subsequently, the cells were covered with 3% bovine serum albumin in PBS (−) at 4 °C for 12 hours to block nonspecific hybridization followed by incubation with an H3K9me3 antibody diluted at 1:100. After washing with PBS (−), the cells were stained by an Alexa 594-conjugated anti-mouse secondary antibody (Molecular Probes, Eugene, OR) diluted at 1: 1,000. Nuclei were counterstained with (DAPI). Fluorescent images were obtained under a TCS SP2 AOBS microscope (Leica, Tokyo, Japan).

### Antitumor efficacy of clomifene in xenograft mouse model *in vivo*

CB-17/Icr-scid mice (male, 4 weeks old) were purchased from HFK Bioscience CO., LTD (Beijing, China). The animals were maintained under ‘specific pathogen-free’ conditions according to the guidelines established by Research Animal Resources, Laboratory Animal Center, Huazhong University of Science and Technology (Wuhan, China). The mice were randomly divided into three groups: vehicle group, 50 or 100 mg/kg clomifene-treated group (n = 6), the experiment was repeated with HT1080 cancer cells. HT1080 cells were inoculated subcutaneously (2 × 10^6^ cells) into the right flank of each mouse in the three groups. Treatment was started after seven days of cells injection. For the clomifene group, 1 or 2 mg clomifene, formulated in 200 *μ*L normal saline, was administered to each mouse every day via gavage. For the vehicle group, 200 *μ*L normal saline was administered to each mouse every day by i.g. The duration of the animal study was 22 days for HT1080 cells. The tumor volume was calculated from measurements of 3 diameters of the individual tumor based on the following formula: tumor volume (mm^3^) = (length × width × height × 0.52). The mice were monitored until tumors reached 1 cm^3^ total volume, at which time the mice were euthanized and the tumors were extracted. The tumors were dissected and sent for immunohistochemical analysis and western blot analysis. All animal experiments were performed following the protocols approved by the Laboratory Animal Center of the Huazhong University of Science and Technology. All animal experiments were performed in accordance with the Guide for the Care and Use of Laboratory Animals of Huazhong University of Science and Technology and approved by the Ethics Committee.

### Statistical analysis

Statistical analysis of the data was performed using Graph Pad Prism 5.0 software. The data were expressed as the means ± SD. Values were analyzed using SPSS version 12.0 software by one-way analysis of variance (ANOVA), and *p* < 0.05 was considered statistically significant.

## SUPPLEMENTARY MATERIALS FIGURES AND TABLES



## Abbreviation

IDH1: Isocitrate dehydrogenase
MST: microscale thermophoresis
CCK8: Cell Counting Kit-8
IPTG: isopropyl *β*-*D*-1-thiogalactopyranoside
PMSF: phenylmethanesulfonyluoride
PVDF: Polyvinylidene
H&E: Hematoxylin-eosin
