# Supplementary material for: Structure based discovery of clomifene as a potent inhibitor of cancer-associated mutant IDH1
Source: Oncotarget. 2017 Apr 27;8(27):44255–65. doi: 10.18632/oncotarget.17464 (PMC5546478; doi:10.18632/oncotarget.17464)
Supplement: Supplementary file 1 [file oncotarget-08-44255-s001.pdf]

Structure based discovery of clomifene as a potent inhibitor of cancer-associated mutant IDH1

SUPPLEMENTARY MATERIALS

SUPPLEMENTARY FIGURES AND TABLES

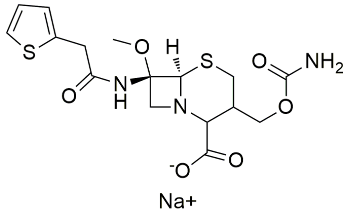

Cefoxitinsodium

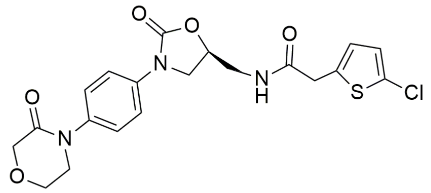

Rivaroxaban

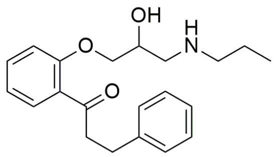

Propafenone

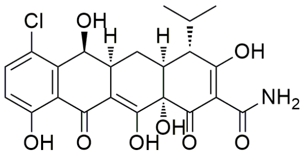

Demeclocycline

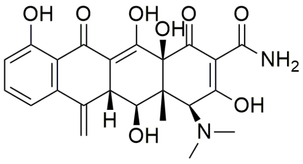

Metacycline

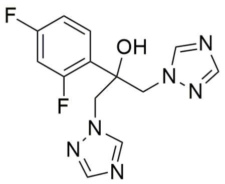

Fluconazole

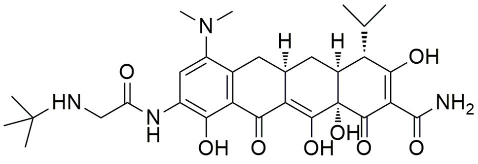

Tigecycline

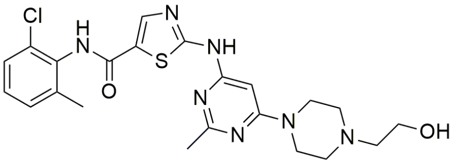

Dasatinib

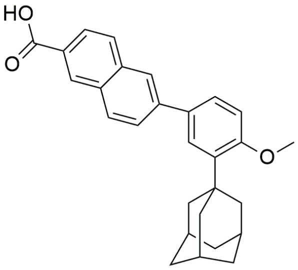

Hydrochloride

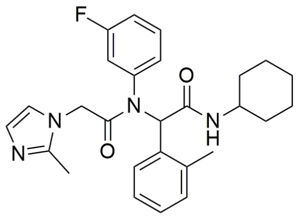

AGI-5198

Supplementary Figure 1: Structures of drugs based on virtual screening.

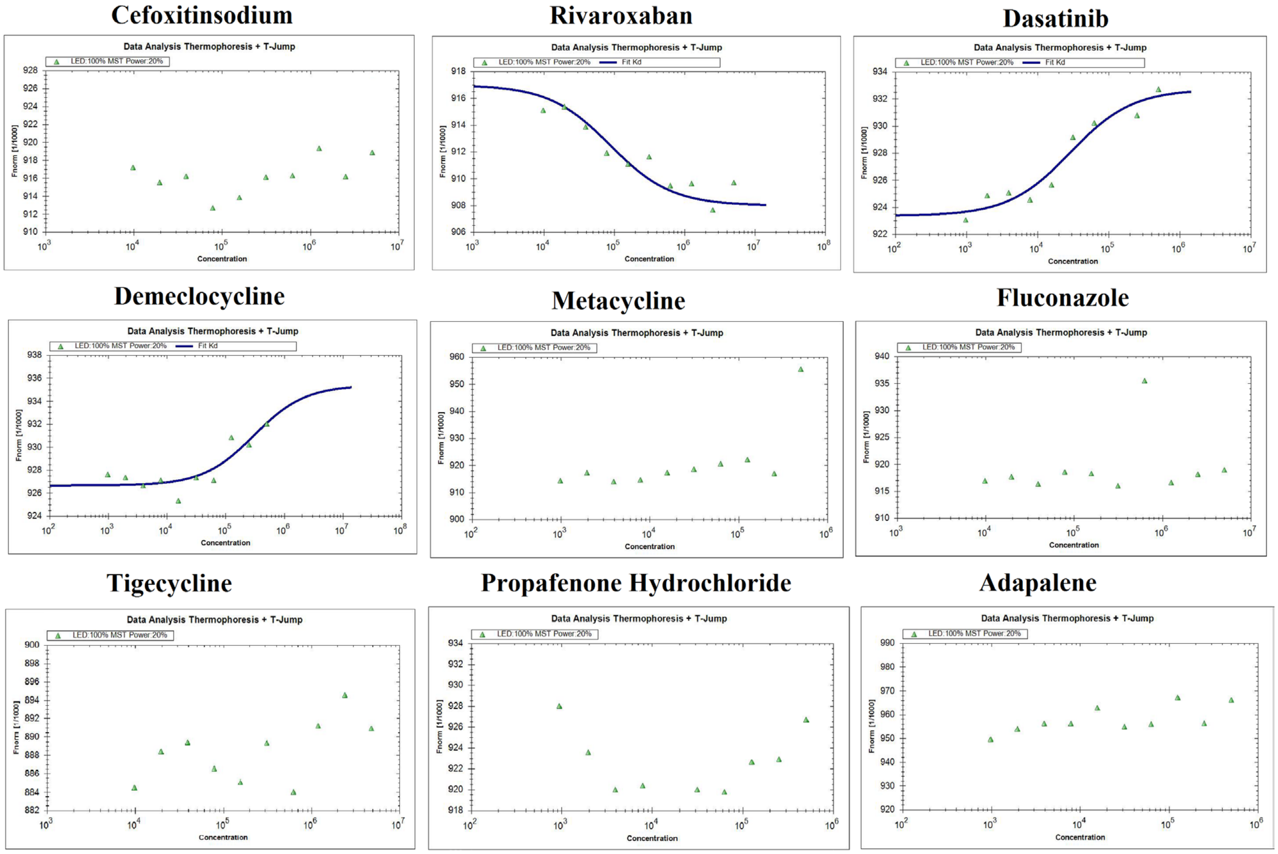

Supplementary Figure 2: Measurement of affinity between virtual screened drugs with IDH1R132H by MST. The resulting binding curves were shown. From the resulting binding curves, Kd of 103.11±11.32 μM for Rivaroxaban, 28.71±2.48 μM for Dasatinib, 303.00±3.15 μM for Demeclocycline were calculated.

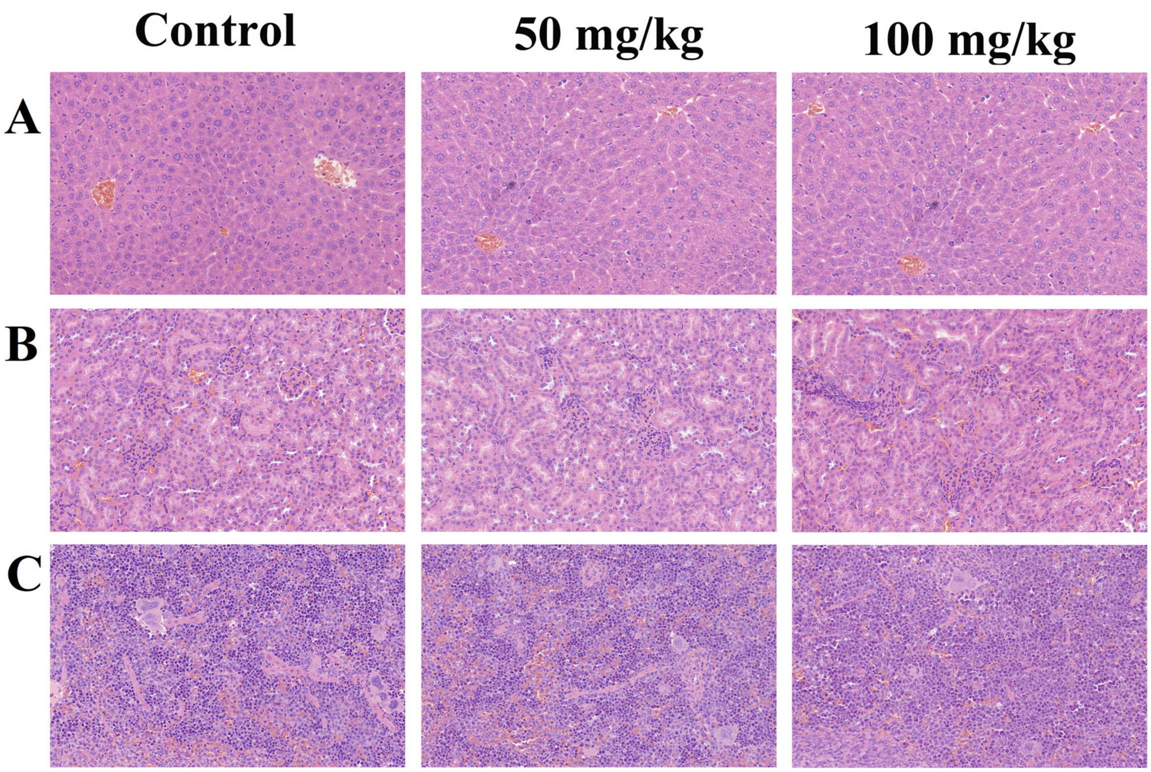

Supplementary Figure 3: Microstructures of sections of the liver, kidney and spleen in control mice and mice treated with doses of clomifene daily for 15 days. No significant damage was observed in all treatment groups. Magnification 400 × (DXIT 1200, Nikon, Japan).

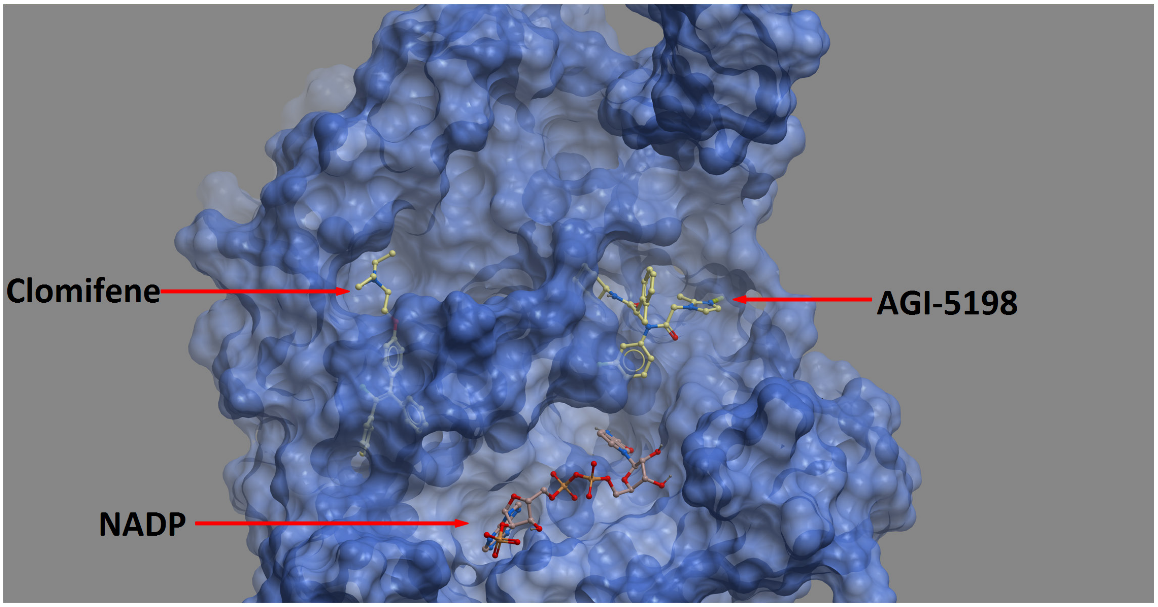

Supplementary Figure 4: Molecular docking predicted that clomifene well fitted the allosteric site of mutant IDH1 with an extended conformation and the binding site of AGI-5198 with mutant IDH1 is close to the pocket of active center.

Supplementary Table 1: Clomifene inhibits IDH1R132H noncompetitively with α-KG

Clomifene (μM)	0	12.5	25	50
Vmax (OD/min)	0.06521	0.05648	0.03804	0.02541
Km (mM)	2.792	3.017	2.298	2.571

Supplementary Table 2: Effects of Clomifene on tumor volume

Group	Model	Clomifene-50	Clomifene-100
Volume(mm^3)	1879.61±244.35	907.73±176.63**	642.91±127.02**
percentage inhibition	0	51.71%	65.80%

Each value was expressed as mean ± S.D., (n=6); *P < 0.05, ** P < 0.01, compared model group.

Supplementary Table 3: Effects of Clomifene on tumor weight

Group	Model	Clomifene-50	Clomifene-100
Weight (g)	2.32±0.93	1.43± 0.42*	0.94 ± 0.61**
percentage inhibition	0	38.36%	59.48%

Each value was expressed as mean ± S.D., (n=6); *P < 0.05, ** P < 0.01, compared model group.

Supplementary Table 4: Effects of Clomifene on R-2HG levels in serum and tumor tissus

Group	Model	Clomifene-50	Clomifene-100
Serum (μM)	5.97 ±2.58	2.89 ±1.06*	1.47 ±1.38**
percentage inhibition	0	51.59%	75.54%
Tissue (μg/g)	364.01 ±118.43	277.34 ±78.03*	155.12 ±74.34**
percentage inhibition	0	23.81%	57.38%

Each value was expressed as mean ± S.D., (n=6); *P < 0.05, ** P < 0.01, compared model group.
